# Ultrafast Airy beam optical parametric oscillator

**DOI:** 10.1038/srep30701

**Published:** 2016-08-01

**Authors:** N. Apurv Chaitanya, S. Chaitanya Kumar, A. Aadhi, G. K. Samanta, M. Ebrahim-Zadeh

**Affiliations:** 1 Photonic Sciences Lab., Physical Research Laboratory, Navarangpura, Ahmedabad 380009, Gujarat, India; 2 Indian Institute of Technology-Gandhinagar, Ahmedabad 382424, Gujarat, India; 3 ICFO-Institut de Ciencies Fotoniques, The Barcelona Institute of Science and Technology, 08860 Castelldefels (Barcelona), Spain; 4 Institucio Catalana de Recerca i Estudis Avancats (ICREA), Passeig Lluis Companys 23, Barcelona 08010, Spain

## Abstract

We report on the first realization of an ultrafast Airy beam optical parametric oscillator (OPO). By introducing intracavity cubic phase modulation to the resonant Gaussian signal in a synchronously-pumped singly-resonant OPO cavity and its subsequent Fourier transformation, we have generated 2-dimensional Airy beam in the output signal across a 250 nm tuning range in the near-infrared. The generated Airy beam can be tuned continuously from 1477 to 1727 nm, providing an average power of as much as 306 mW at 1632 nm in pulses of ~23 ps duration with a spectral bandwidth of 1.7 nm.

Mathematical resemblance of paraxial wave equation to the free-particle Schrödinger wave equation with Airy wave packet as one of the solutions^1^ enabled the prediction^2^ and demonstration^3^ of optical beams having transverse intensity distribution described by the Airy function. Unlike other structured beams, Airy beams have peculiar characteristics such as beam shape invariance with propagation, “diffraction-free” propagation along curved trajectories in free space, “self-acceleration” and self-restoration of beam shape even after obstruction by small objects, and “self-healing”. Since the first experimental demonstration^3^, the Airy beam has attracted a great deal of attention for potential applications in diverse areas including optical routing^4^, manipulation of microscopic particles^5^,^6^, optically mediated particle clearing^7^, and laser micromachining^8^. Additionally, propagation characteristics of the Airy beam in nonlinear^9^,^10^,^11^,^12^,^13^,^14^,^15^ and turbulent^16^ media have been studied for the generation of curved plasma channel^11^, supercontinuum and solitary wave^12^,^13^, and laser filamentation^14^,^15^. Efforts have also been made to demonstrate Airy beam with electron waves^17^, acoustic waves^18^, and surface plasmon polaritons^19^.

Many of the applications and studies such as laser micromachining of curve surfaces, laser filamentation, supercontinnum generation and curved plasma channel require high-power ultrafast Airy beams with suitable spectral and temporal parameters at different wavelengths across the electromagnetic spectrum. Conventionally, the Airy beam is generated through cubic phase modulation of a laser beam in Gaussian intensity distribution and its subsequent Fourier transformation^3^. However, the Airy beams so far generated cannot provide high power and wide spectral coverage from a single system. For the generation of high-power Airy beam with wide wavelength tunability^20^, we have recently explored the intrinsic tuning capability and high intracavity power of a continuous-wave (cw) optical parametric oscillator (OPO) in singly resonant oscillator (SRO) design^21^. Here, we demonstrate, for the first time to our knowledge, a new class of Airy beam source based on an ultrafast OPO. The source is realized by cubic phase modulation and subsequent Fourier transformation of the intracavity resonant signal of a synchronously-pumped SRO, producing output pulses of 23 ps duration in 2-D Airy intensity distribution, with an average power of as much as 306 mW at 78 MHz repetition rate, and tunable over 250 nm across 1477–1727 nm in the near-infrared.

## Theoretical Background

The two-dimensional (2-D) intensity distribution of a finite-energy Airy beam can be expressed in the form,


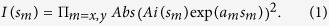


Here, *Ai*(*s*_*m*_) is the Airy function^22^, *s*_*x*_ = *x*/*x*_0_ and *s*_*y*_ = *y*/*y*_*0*_ are the normalized transverse coordinates along *x* and *y* axis, respectively, and *x*_0_, *y*_*0*_ are the transverse scaling (or characteristic) parameters, and *a* is the truncation parameter, which defines the extent of Airy beam in 2-D plane (*x-y* plane). The parabolic trajectory of the Airy beam of wavelength, λ, along the *x* and *y* axis, as the beam propagates along the *z* axis, is determined by the characteristic parameters, *x*_0_, *y*_*0*_, and the launching angles, *θ*_*x*_ and *θ*_*y*_, through the relation, *m*_*d*_ = *d*_*m*_*z*^2^+*θ*_*m*_*z*, where *m* *=* *x, y*, and 
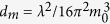
. For the special case where *x*_0_ = *y*_*0*_ (symmetric Airy beam), the transverse acceleration in the *x*-*y* plane can be expressed as^2^


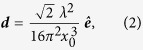


where 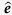 is a unit vector in the *x*-*y* plane along the direction of acceleration, which is determined by the relative orientation of the beam in the *x-y* plane. In this case, *x*_0_ = *y*_*0*_, the parabolic trajectory with respect to 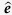 along the propagation is given by


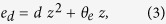


where *θ*_*e*_ is the angle the beam propagation vector makes with respect to the unit vector 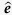.

To generate finite-energy Airy beam, we modulated the phase and amplitude of the Gaussian beam using a cubic phase mask in the form of a binary diffraction grating with amplitude transmittance, as^20^


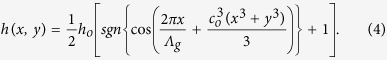


Here, *h*_*o*_ is the ridge height of the grating with period, Λ_g_, defined by the ratio of the number of lines, *N*, along the width, *L,* of the grating in *x* direction. The constant, *c*_*o*_, represents the strength of cubic phase modulation in the transverse direction. The phase of the 1^st^-order diffracted beams can be represented as


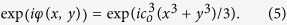


## Experiment

**Figure 1:**
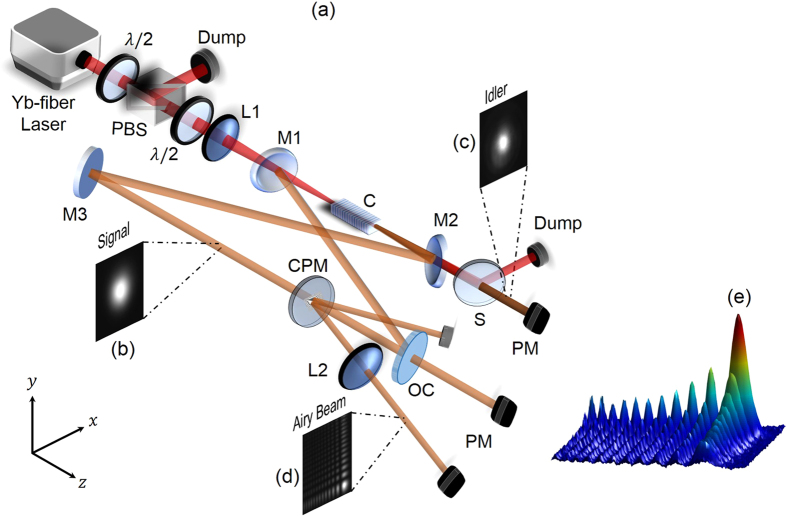
Ultrafast Airy beam optical parametric oscillator. (**a**) Schematic illustration of the experimental setup for the ultrafast Airy beam OPO. λ/2: half-wave plate; PBS: polarizing beam-splitter cube; L1-2: Lenses; M1-3: mirrors; OC: output coupler; C: MgO:PPLN crystal (in oven); CPM: cubic phase mask; S: dichroic mirrors; PM: power meter. Recorded intensity profile of (**b**) resonant signal, (**c**) idler, and (**d**) Airy beam, (**e**) 3-D illustration of the generated Airy beam.

The schematic of the experimental setup for the ultrafast Airy beam OPO is shown in Fig. 1(a). A mode-locked picosecond Yb-fiber laser (Fianium, FP1060-20) providing a maximum average power of 20 W at 1064 nm is used to synchronously pump the Airy beam SRO. The output has a full-width at half-maximum (FWHM) spectral bandwidth of 1.4 nm in pulses of 20 ps duration at 78 MHz repetition rate. The laser is operated at full power, and the input power to the SRO is controlled using a combination of a half-wave (λ/2) plate and a polarizing beam-splitter cube. A second λ/2 plate is used for control of the input pump polarization for phase-matching in the OPO crystal. A lens, L1, of focal length, *f *= 175 mm, is used to focus the pump beam at the center of the 50-mm-long, 8.6-mm-wide and 1-mm-thick, multi-grating MgO:PPLN crystal (C), used as the gain medium for the OPO. The crystal has 7 channels with quasi-phase-matching (QPM) grating periods ranging from Λ = 28.5 to 31.5 *μ*m in steps of 0.5 *μ*m, with both faces antireflection (AR)-coated over 1400–2000 nm and at 1064 nm. The crystal is housed in an oven, which can be adjusted from room temperature to T = 200 °C in steps of 0.1 °C. The OPO is designed in a four-mirror ring cavity with two plano-concave mirrors (M1-2) of radius of curvature (*ROC *= 100 mm), plane mirror, M3, and a plane output coupler (OC). The cavity length is perfectly synchronized to the repetition rate of the pump laser. All mirrors, M1–3, are coated for high reflectivity for the signal (*R*>99%) over 1300–1900 nm, and high transmission for the idler (*T*>80%) over 2200–4000 nm and the pump (*T*>90%) at 1064 nm, thus ensuring SRO operation. The OC has a partial transmission (*T* ~ 5%) over 1100–1630 nm. A custom-designed AR-coated cubic phase mask (CPM) in the form of binary grating (Holo-OR), fabricated at the center of a 3-mm-thick fused silica substrate of diameter 25.4 mm over a region of 2 × 2 mm^2^ with phase modulation given by Eq. 4, is introduced inside the SRO cavity between mirror M3 and OC, to modulate amplitude and phase of the resonant Gaussian beam in the first-order diffracted beams. The binary grating in the phase mask has *N *= 100 lines across the *L *= 2 mm width, resulting in a carrier period of Λ_g_ = 20 μm. The ridge height, *h*_*o*_, is optimized to provide 0^th^ transmission of ~95% at 1500 nm, resulting in ~5% coupling of the intracavity signal into the Airy beam the first diffraction order. It is to be noted that the higher diffraction orders have negligibly small intensity. The CPM has cubic phase modulation strength estimated to be, *c*_*o*_ = 5.77/mm^20^. A lens, L2, of focal length, *f *= 500 mm, is used for Fourier transformation of this out-coupled beam from the OPO into the Airy beam.

## Discussions

**Figure 2:**
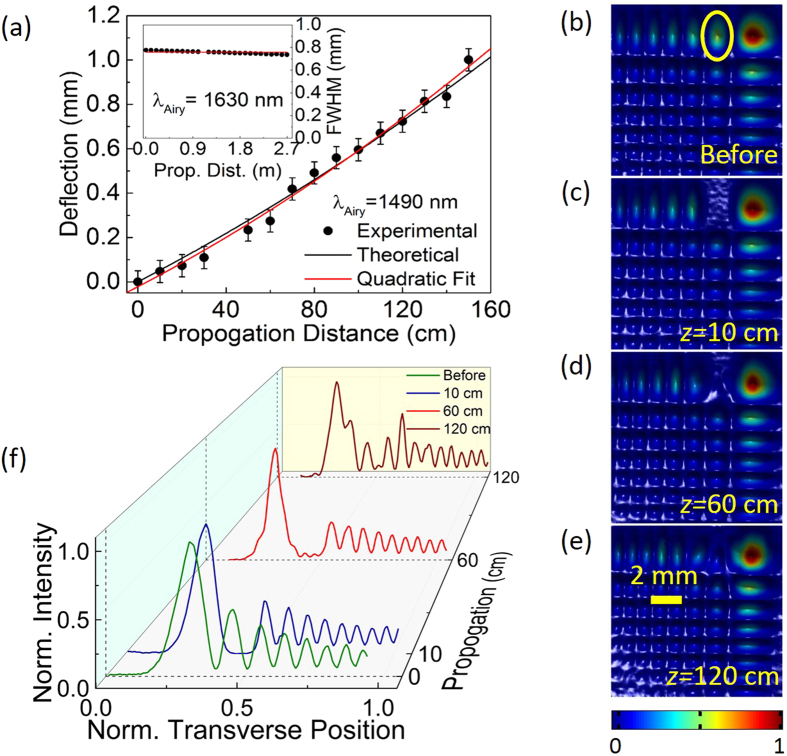
Characteristics of Airy beam. (**a**) Measurement of beam acceleration of the Airy beam OPO as a function of propagation distance. Solid line (black) represents theoretically predicted acceleration, (red) is the quadratic fit to the experimental data (dots). (Inset) Variation in the width (FWHM) of the central lobe of the Airy beam over propagation. Solid line (red) is linear fit to data (dots). (**b**–**e**) Verification of self-healing property of the output beam of the Airy beam OPO. (**f**) The line profile of the beam at a distance *z* = 10 cm (blue), *z* = 60 cm (red), *z *= 120 cm (brown) from the Fourier plane.

The output intensity profile at the focus of the Fourier transforming lens, L2, recorded using a CCD-based camera, is shown in Fig. 1(d,e), depicting the intensity profile of the generated beam in 3-D. The recorded intensity profile clearly confirms the generation of 2-D Airy beam. The parameters, *x*_0_, *y*_0_, *a*_*x*_, *a*_*y*_, of the generated beam were determined by fitting the intensity profile with a 2-D Airy function, as represented by Eq. (1). Although the intensity distribution in Fig. 1(d) appears to be an Airy beam, nevertheless, to confirm the generation of Airy beam, we investigated some of its intriguing properties such as acceleration, non-diffraction, and self-healing. To study self-acceleration of the generated beam across the SRO tuning range, we recorded the intensity profile as a function of propagation distance at an arbitrary wavelength (*λ*_*Airy*_ = 1490 nm), and measured the position of the beam (central lobe). The results are shown in Fig. 2(a). As can be seen, the beam deflects from its rectilinear path by a distance of ~1 mm over a propagation distance of 1.5 m. Using the intensity profiles, we measured the transverse scaling parameters, *x*_0_ and *y*_0_, to be ~0.65 mm, confirming the generation of a symmetric Airy beam. Once the *x*_0_ parameter of the beam is known, apart from an angular offset given by *θ*_*e*_, the trajectory of the Airy beam can be predicted theoretically using Eq. (3). Fitting a second-degree polynomial (solid red line) to the experimental data points in Fig. 2(a), the angular offset, *θ*_*e*_, is estimated to be 0.5 mrad. For the given set of *x*_0_ and *θ*_*e*_, the theoretically predicted transverse acceleration can be obtained, as shown by the solid black line in Fig. 2(a). As evident from the plot, the experimentally observed acceleration of ~1 mm is in close agreement with the theoretically predicted value of ~0.94 mm. We also observed self-acceleration of the Airy beam across the tuning range, with lower acceleration at longer wavelengths. This is attributed to the fact that the scaling parameters, *x*_0_ and *y*_0_, of the Airy beam are linearly proportional to its wavelength^20^. To verify non-diffraction property of the Airy beam, we measured the full-width at half-maximum (FWHM) linewidth of the central lobe of the beam at a wavelength, *λ*_*Airy*_ = 1630 nm, over a propagation distance of 2.7 m, with the results shown in the inset of Fig. 2(a). The width of the central lobe varies from 0.77 ± 0.05 mm at *z *= 0 to 0.73 ± 0.05 mm at *z *= 2.7 m. Such observation clearly shows that, within the experimental error, the beam remains diffraction-free (propagation invariant) over >2.7 m.

We also investigated the self-healing nature of the output beam from the Airy beam OPO at a signal wavelength of *λ*_*Airy*_ = 1630 nm, with the results shown in Fig. 2(b–f). At a distance, *z *= 10 mm, from the focal point of the Fourier lens, L2, we blocked one of the lobes [circled region in Fig. 2(b)] of the output beam using a knife edge and recorded the intensity distribution of the beam over a propagation distance in free space. As evident from Fig. 2(c–e), the Airy beam has no second lobe along *x* axis of Fig. 2(c) at *z *= 10 cm. However, at *z *= 60 cm, we observe the beam intensity re-appears in the blocked region, with a complete reproduction of the beam shape at propagation distance, *z *= 120 cm, as shown in Fig. 2(e). These results clearly confirm the self-healing nature of the generated beam corresponding to an Airy beam. Fig. 2(f) shows the line profile of the Airy beam intensity along the propagation direction for better understanding of the self-healing property.

**Figure 3:**
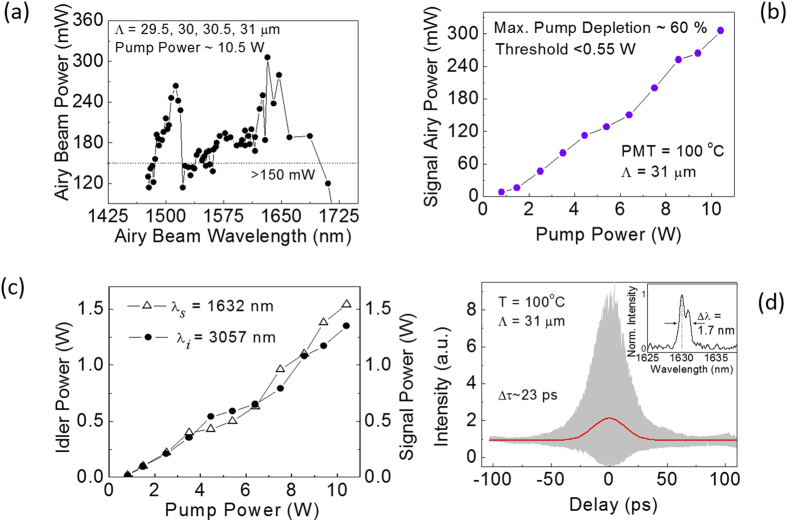
Performance of the ultrafast Airy beam source. (**a**) Variation of the Airy beam signal power across the tuning range of the ultrafast OPO, while pumping with ~10.5 W of input power. (**b**) Variation of Airy beam (λ_Airy_ = 1632 nm) signal power with pump power. (**c**) Dependence of signal (λ_s_ = 1632 nm) and idler (λ_i_ = 3057 nm) power in Gaussian intensity distribution on pump power. (**d**) Interference autocorrelation trace of the Airy beam signal pulses. Inset: Corresponding spectrum.

After confirming the generation of the Airy beam from the OPO, we characterized the oscillator with regard to its output parameters. Using the QPM grating periods, Λ = 29.5, 30, 30.5 and 31 *μ*m, in the MgO:PPLN crystal, and varying the phase-matching temperature, we tuned the signal wavelength of the Airy beam OPO continuously from 1477 nm to 1727 nm. In addition, the OPO provides corresponding idler radiation from 3805 nm to 2771 nm in Gaussian intensity distribution. Since conventional MgO:PPLN picosecond OPOs can afford significant output coupling losses^23^, we used an OC (*T* ~ 5%) to extract the resonant signal beam from the oscillator. The signal output power in the Airy beam across the tuning range with the ~5% OC, while pumping with an input power of ~10.5 W, is shown in Fig. 3(a). As can be seen, we were able to extract an output power >150 mW in the Airy beam signal over >85% of the full tuning range, with a maximum power of 306 mW obtained at *λ*_*Airy*_ = 1632 nm. The lower value of the Airy beam signal power can be attributed to the reduced diffraction efficiency (~5%) of the CPM. However, the output power of the signal radiation in Gaussian beam profile out-coupled through the OC (*T* ~ 5%) varies in the range of 0.6 to 2.5 W across the tuning range. Additionally, the idler radiation in Gaussian beam profile has similar output power as that of the out-coupled signal across the tuning range. The higher out-coupled signal power in Gaussian beam profile as compared to that in the Airy beam profile suggests the possibility of further improvement in the Airy beam power with optimization of CPM grating for enhanced diffraction efficiency across the tuning range.

To measure the power-scaling behavior of the ultrafast Airy beam source, we recorded the Airy beam power at *λ*_*Airy*_ = 1632 nm (T = 100 °C, Λ = 31 μm), while varying the input pump power to the OPO. The results are shown in Fig. 3(b), where it can be seen that the Airy beam power increases almost linearly with the pump power, with no signs of saturation. The maximum signal Airy beam power is 306 mW for 10.5 W of input pump power, as expected. We also measured the variation in the power of out-coupled Gaussian signal (λ_s_ = 1362 nm) and corresponding idler (λ_i_ = 3057 nm) beams with the pump power, with the result shown in Fig. 3(c). The Gaussian signal (idler) can provide a maximum average power of 1.54 W (1.35 W). The SRO has a threshold of <0.55 W with a maximum pump depletion of ~60%.

We also performed temporal and spectral characterization of the output signal Airy beam. Operating the Airy beam source at λ_Airy_ = 1632 nm, we measured the temporal duration of the Airy beam pulses using a home-made interferometric autocorrelator. The result is shown in Fig. 3(d), where a pulse width of Δτ ~ 23 ps (assuming Gaussian temporal profile) is obtained. The simultaneously measured spectral bandwidth of the Airy beam, shown in the inset of Fig. 3(d), is Δλ = 1.7 nm (FWHM), resulting in a time-bandwidth product of ΔτΔν = 4.4, well above the transform limit in the absence of dispersion management in the OPO cavity.

In conclusion, we have experimentally demonstrated the first ultrafast Airy beam OPO. We have confirmed the generation of Airy beam by verifying its characteristic properties with regard to self-acceleration, non-diffraction and self-healing. The Airy beam OPO produces an average power of as much as 306 mW with a tunable coverage across 1477–1727 nm. Using optimized diffraction efficiency of the cubic phase grating, we can further increase the output power in the Airy beam across the tuning range. The source also produces Gaussian signal (idler) beam with average power of up to 1.35 W (1.54 W) for a pump power of 10.5 W. The Airy beam has temporal and spectral bandwidth of Δτ ~ 23 ps and Δλ** **=** **1.7** **nm, respectively, which can be improved to approach the transform limit with dispersion control of the OPO cavity.

## Additional Information

**How to cite this article**: Apurv Chaitanya, N. *et al*. Ultrafast Airy beam optical parametric oscillator. *Sci. Rep.* **6**, 30701; doi: 10.1038/srep30701 (2016).
